# PCSK9 Inhibitors “Fast Track” Use Versus “Stepwise” Lipid-Lowering Therapy in Patients with Acute Coronary Syndrome: A Retrospective Single-Center Study in a “Real-World” Population

**DOI:** 10.3390/jcm14092992

**Published:** 2025-04-26

**Authors:** Davide D’Andrea, Valentina Capone, Alessandro Bellis, Rossana Castaldo, Monica Franzese, Gerardo Carpinella, Fulvio Furbatto, Fulvio La Rocca, Fabio Marsico, Raffaele Marfella, Giuseppe Paolisso, Pasquale Paolisso, Carlo Fumagalli, Maurizio Cappiello, Eduardo Bossone, Ciro Mauro

**Affiliations:** 1 Unità Operativa Complessa Cardiologia con UTIC ed Emodinamica, Dipartimento Reti Tempo-Dipendenti, Azienda Ospedaliera “Antonio Cardarelli”, Via A. Cardarelli n. 9, 80131 Napoli, Italy; davide.dandrea@aocardarelli.it (D.D.); valentina.capone@aocardarelli.it (V.C.); gerardo.carpinella@aocardarelli.it (G.C.); fulvio.furbatto@aocardarelli.it (F.F.); fulvio.larocca@aocardarelli.it (F.L.R.); fabio.marsico@aocardarelli.it (F.M.); ciro.mauro@aocardarelli.it (C.M.); 2 Bioinformatics Lab, SDN-SYNLAB, IRCCS SDN Spa, 80143 Napoli, Italy; rossana.castaldo@synlab.it (R.C.); monica.franzese@synlab.it (M.F.); 3 Department of Medical, Surgical, Neurological, Aging and Metabolic Sciences, University of Campania “Luigi Vanvitelli”, Piazza Miraglia, 2, 80138 Napoli, Italy; raffaele.marfella@unicampania.it (R.M.); giuseppe.paolisso@unicampania.it (G.P.); pasquale.paolisso@unicampania.it (P.P.); carlo.fumagalli@unicampania.it (C.F.); 4 Department of Health Area Strategic Services, Azienda Ospedaliera “Antonio Cardarelli”, Via A. Cardarelli n. 9, 80131 Napoli, Italy; maurizio.cappiello@aocardarelli.it; 5 Department of Advanced Biomedical Sciences, Federico II University Hospital, Via S. Pansinin. 5, 80131 Napoli, Italy; eduardo.bossone@unina.it

**Keywords:** PCSK9i, dyslipidemia, “fast track” strategy, acute cardiovascular syndrome, LDL-C

## Abstract

**Background**: The “fast track” addition (within 48 h) of proprotein convertase subtilisin/kexin type 9 inhibitors (PCSK9i) to the optimized oral lipid-lowering therapy (LLT) during hospitalization for acute coronary syndrome (ACS) has been shown to rapidly achieve the low-density lipoprotein cholesterol (LDL-C) therapeutic targets. However, so far, its efficacy in real-world settings remains understudied. **Methods**: We retrospectively analyzed 128 ACS patients treated at our center, comparing “PCSK9i fast track” use within 48 h to standard “stepwise” LLT. Lipid levels and incidence of major adverse cardiovascular events (MACEs) were evaluated at 30 and 180 days. **Results**: The “PCSK9i fast track” group achieved significantly lower LDL-C levels at 30 days (41.5 ± 27.5 vs. 85.6 ± 35.9 mg/dL, *p* < 0.001) and 180 days (29.6 ± 21.0 vs. 59.0 ± 32.4 mg/dL, *p* < 0.001). Recommended LDL-C targets (<55 mg/dL) were met by 88.3% of the “PCSK9i fast track” group at 180 days, compared with 61.9% of controls (*p* < 0.001). No significant differences in MACEs were observed between groups. No adverse effects from PCSK9i use were noted. **Conclusions**: The “PCSK9i fast track” strategy was safe and effective in achieving LDL-C targets more rapidly than conventional approaches in real-world ACS patients.

## 1. Introduction

The current management of acute coronary syndrome (ACS) has achieved a remarkable improvement in outcomes through the wide diffusion of percutaneous coronary interventions (PCIs) and pharmacological therapies that aim to reduce the occurrence of coronary restenosis and myocardial reperfusion injury. However, the rate of mortality in these patients remains high [1].

The choice of an aggressive pharmacological approach to decrease the low-density lipoprotein cholesterol (LDL-C) values in patients with recent ACS (1 to 12 months earlier) has allowed us to reduce the incidence of major adverse cardiovascular events (MACEs) [2,3]. Proprotein convertase subtilisin/kexin type 9 (PCSK9) inhibitors (PCSK9i) have been shown to play a pivotal role to address this aim, and their use during hospitalization for ACS was first suggested in the 2019 ESC guidelines for patients with LDL-C >100 mg/dL despite having already taken the maximum tolerated statin dosage plus ezetimibe for 4–6 weeks (Class IIa, Level of Evidence C) [4]. Nevertheless, a survey designed to compare post-ACS patient management in 2022 with that in 2018 revealed that, while LDL-C goal achievement has improved since the release of the 2019 ESC guidelines, lipid management in post-ACS patients has remained suboptimal [4]. Similarly, in an Italian real-world registry of ACS patients undergoing a PCI, PCSK9is prescription was limited to 10% of cases and the therapeutic LDL-C target was only obtained in 62% of the enrolled subjects [5]. Thus, a “change in the paradigm” seemed necessary in this setting.

In 2022, a clinical consensus statement by the Association for Acute Cardiovascular Care proposed to consider PCSK9i in the acute phase of ACS, independently of LDL-C profile, if additional high-risk features (such as multivessel cardiovascular disease, diabetes mellitus, multivascular disease, familiar hypercholesterolemia, and recurrent ischemic cardiovascular events) are present [6]. Consistently, in the same year, the “Agenzia Italiana del Farmaco” (AIFA) allowed PCSK9i use in LLT-naïve patients presenting LDL-C > 140 mg/dL and lowered the LDL-C threshold for PCSK9i administration to values > 70 mg/dL in patients already taking optimized oral LLT during hospitalization for ACS [7]. In line with these indications, the 2023 ESC guidelines for the management of ACS recommended the addition of PCSK9i during hospitalization for ACS (Class I, Level of Evidence A) in patients with LDL-C > 55 mg/dL, despite the optimized oral LLT [8].

This switch from the concept “the lower, the better” to “strike early, strike strong” has prompted the testing of immediate PCSK9i addition to optimal oral LLT during hospitalization for ACS, even independently of LDL-C baseline values and clinical risk profile [9,10,11]. The “PCSK9i fast track” strategy has demonstrated the ability to achieve the LDL-C therapeutic target within 1 month in a higher percentage of patients compared with the “stepwise” approach suggested by the ESC guidelines in randomized clinical trials [9,10,11,12,13]. Nevertheless, this strategy has not yet been tested in a real-world population.

The primary endpoint of our study was to retrospectively compare the effect of the “PCSK9i fast track” strategy with optimized oral LLT alone on the lipid profile in a cohort of ACS patients referred to the coronary care unit of “Antonio Cardarelli” Hospital, Naples. As a secondary endpoint, the incidence of MACEs (a composite of death for any cause, cardiac death, recurrent ACS, ischemia-driven revascularization, and new PCI revascularization) was analyzed.

## 2. Methods

### 2.1. Study Population

**Figure 1:**
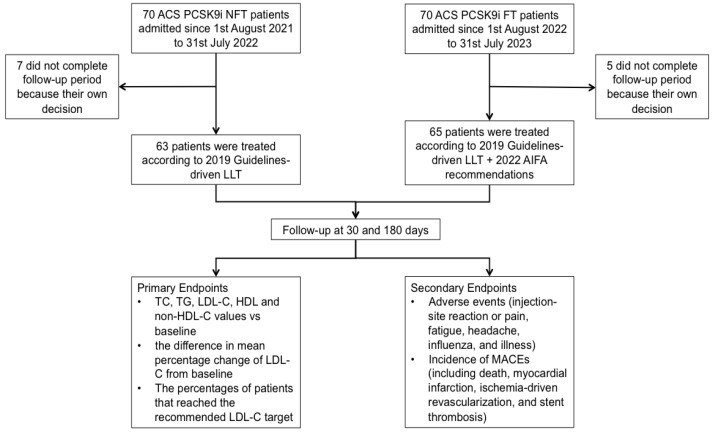
Flow diagram of the study. AIFA, Agenzia Italiana del Farmaco; FT, fast track; LDL-C, low-density lipoprotein cholesterol; LLT, lipid-lowering therapy; NFT, non-fast track; non-HDL, non-high-density lipoprotein; TC, total cholesterol; TG, triglycerides.

A retrospective single-center study was conducted comparing 70 patients (cases), admitted to our Cardiological Department from 1 August 2022 to 31 July 2023 with a diagnosis of ACS, that were included in the “PCSK9i fast track” group (“PCSK9i FT”), with a group of 70 patients (controls), admitted to our Cardiological Department from 1 August 2021 to 31 July 2022 with a diagnosis of ACS, that were included in the control group (i.e., the “guideline-driven LLT” group), called the PCSK9i non-fast track (“PCSK9i NFT”) group. Five patients in the “PCSK9i FT” group and seven controls did not complete the follow-up because of their own decision. Therefore, the final study population consisted of 65 patients in the PCSK9i FT group and 63 patients in the PCSK9i NFT group (i.e., the “guideline-driven LLT” group; Figure 1).

### 2.2. Study Treatment

The patients included in the “PCSK9i FT” group were treated according to the 2019 ESC guidelines for the management of dyslipidemias and the 2022 indications from the AIFA [7,14]. They received a single subcutaneous dose of evolocumab 140 mg or alirocumab 150 mg, in addition to the optimized oral LLT, within 48 h after ACS diagnosis, if LDL-C values were >140 mg/dL in the absence of a previous LLT or if LDL-C values were >70 mg/dL in presence of a previous optimized oral LLT. The following doses of evolocumab 140 mg or alirocumab 150 mg were administered every two weeks.

The patients included in the “PCSK9i NFT” group were treated with the “stepwise” LLT recommended by the 2019 ESC guidelines for the management of dyslipidemias [8]. Thus, the LLT-naïve patients belonging to this group began to receive the association of statin at the maximum tolerated dosage plus ezetimibe, and the PCSK9i was added during the follow-up period if the LDL-C target had not been achieved after 4–6 weeks. In the same group, the PCSK9i was immediately prescribed to those patients already on optimized oral LLT before ACS if LDL-C levels were >100 mg/dL.

The choice between evolocumab and alirocumab was at the discretion of the cardiologist. The follow-up for both the groups was performed at 30 and 180 days (Figure 1).

### 2.3. Study Endpoints

Clinical (CV risk factors, previous ACS or coronary revascularization, symptoms), biochemical (glucose blood levels at admission and at discharge), echocardiographic (left ventricular ejection fraction or LVEF at admission and at discharge), and angiographic (PCI-related culprit vessels, the proximal localization of stenosis, and stent length) characteristics of both groups were detected. To identify the proximal segment for each major epicardial coronary artery, we referred to the definition by Serruys et al. [15]. More specifically, (1) proximal right coronary artery (proximal RCA): from the ostium to one half the distance to the acute margin of the heart; (2) proximal left anterior descending (proximal LAD): from proximal to and including the first major septal branch; and (3) circumflex artery (proximal Cx): the main stem of the Cx from its origin of the left main and including the origin of the first obtuse marginal branch.

Plasma levels of total cholesterol (TC), high-density lipoprotein cholesterol (HDL-C), triglycerides (TG), LDL-C, and non-HDL-C were detected at admission to the Emergency Department and at 30 and 180 days of follow-up. The percentage of patients in the “PCSK9i FT” and “PCSK9i NFT” groups that reached the recommended target after 30 and 180 days was calculated (Figure 1).

Data on the safety of early PCSK9i administration and adherence to PCSK9i therapy are also reported.

Finally, the incidence of MACEs, including death, myocardial infarction, ischemia-driven revascularization, and stent thrombosis, was investigated both during hospitalization and the follow-up period (Figure 1).

### 2.4. Statistical Analysis

Statistical analysis was performed using R Core Team (version 3.03, Austria). Continuous variables are expressed as the mean and standard deviation (SD). The data distribution was tested for normality through the Shapiro–Wilk test. An unpaired Student’s *t*-test or the Wilcoxon rank-sum test, as required, was used for comparison between two groups. For comparison among more than two groups, ANOVA or the Kruskal–Wallis test was used. Categorical variables are expressed as a percentage and were compared using the Chi-Square test or Fisher’s exact test. A *p*-value of <0.05 was considered significant. Bonferroni’s correction was used for multiple hypothesis correction if necessary. Boxplots were generated only for features that significantly changed (*p*-value < 0.05).

## 3. Results

Most features were non-normally distributed by the Shapiro–Wilk test. Therefore, non-parametric tests were used, such as the Wilcoxon rank-sum test, to investigate the difference between two groups and, in the case of more than two groups, the Kruskal–Wallis test was employed. Statistical analysis for categorical variables between controls and cases is reported in Table 1. The “PCSK9i FT” and “PCSK9i NFT” groups did not significantly differ in sex, diabetes mellitus, smoking habits, and number of LLT-naïve patients (*p*-value > 0.05; Table 1). Conversely, they significantly did in prevalence of familiarity for CAD and arterial hypertension (*p* < 0.05). Unstable angina was the admission diagnosis in only four cases in the “PCSK9i FT” group. No statistically significant differences were observed in angiographic characteristics between the “PCSK9i FT” and “PCSK9i NFT” groups.

Statistical analysis for continuous variables between controls and cases is reported in Table 2. The “PCSK9i FT” and “PCSK9i NFT” groups did not significantly differ in body mass index (BMI), stent total length, LVEF at admission and at discharge, and glucose blood levels at admission and at discharge. Conversely, they significantly differed in age (*p*-value < 0.05) and in stent maximum diameter (*p*-value < 0.05). At hospital admission (day 0), TC, LDL-C, HDL-C, TGs, and non-HDL-C did not differ between the two groups (*p*-value > 0.05).

In the “PCSK9i FT” group, 26 patients (40%) were treated with evolocumab and 39 patients (60%) were treated with alirocumab, while in the “PCSK9i NFT” group 5 patients (8%) initiated therapy with evolocumab and 10 patients (16%) initiated therapy with alirocumab during the follow-up period. All of the remaining 48 patients (74%) in the “PCSK9i NFT” group were never prescribed PCSK9is because they achieved the LDL-C target according the 2019 ESC guidelines for the management of dyslipidemias with the maximum tolerated statin dosage plus ezetimibe. The compliance with the PCSK9i treatment was 100% for both groups. This result was achieved thanks to the establishment of a dedicated ambulatory for the prescription of PCSK9is.

**Figure 2:**
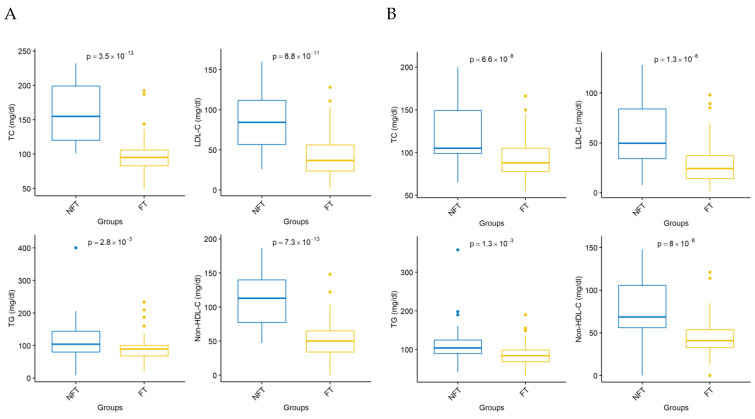
Comparison of lipid values between the “PCSK9i FT” and “PCSK9i NFT” groups at 30 days and 180 days. Total cholesterol, low-density lipoprotein cholesterol, triglycerides, and non-high-density lipoprotein cholesterol values were significantly lower (*p* < 0.05) in the “PCSK9i FT” group compared with the “PCSK9i NFT” group (*p* < 0.05), both at 30 days (**A**) and 180 days (**B**) of follow-up. FT, fast track; LDL-C, low-density lipoprotein cholesterol; NFT, non-fast track; non-HDL, non-high-density lipoprotein cholesterol; TC, total cholesterol; TG, triglycerides.

Both at 30 and 180 days of follow-up, TC, TG, LDL-C, and non-HDL-C values were significantly lower in the “PCSK9i FT” group than in the “PCSK9i NFT” group (*p*-value < 0.05), whereas HDL-C values at 180 days were higher in the cases than in the controls (*p*-value < 0.05; Figure 2A,B).

**Figure 3:**
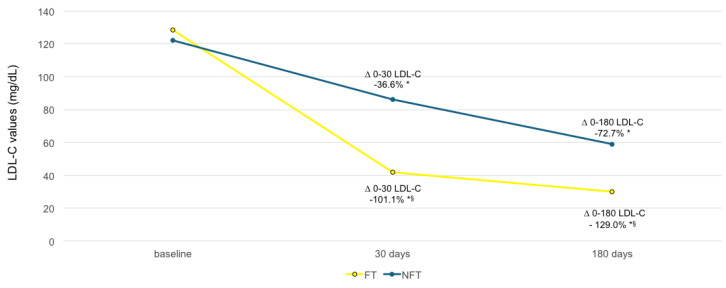
Difference in the mean percentage change of LDL-C from baseline to follow-up and between the “PCSK9i NFT” and “PCSK9i FT” groups at 30 and at 180 days. At 30 days of follow-up, the difference in the mean percentage change of LDL-C from baseline to follow-up (Δ LDL-C) was −101.1% in the “PCSK9i FT” group versus −36.6% in the “PCSK9i NFT” group (*p*-value < 0.001), whereas at 180 days the difference in the mean percentage change of LDL-C from baseline to follow-up (Δ LDL-C) was −129.0% in the “PCSK9i FT” group versus −72.7% in the “PCSK9i NFT” group (*p*-value < 0.001). FT, fast track; LDL-C, low-density lipoprotein cholesterol; NFT, non-fast track. * *p* < 0.001 versus baseline; ^§^ *p* < 0.001 versus the “PCSK9i NFT” group.

At 30 days of follow-up, the difference in the mean percentage change of LDL-C from baseline to follow-up was −101.1% in the “PCSK9i FT” group versus -36.6% in the “PCSK9i NFT” group (*p*-value < 0.001, versus baseline and between group difference), whereas at 180 days the difference in the mean percentage change of LDL-C from baseline to follow-up was −129.0% in the “PCSK9i FT” group versus −72.7% in the “PCSK9i NFT” group (*p*-value < 0.001, versus baseline and between group difference; Figure 3).

**Figure 4:**
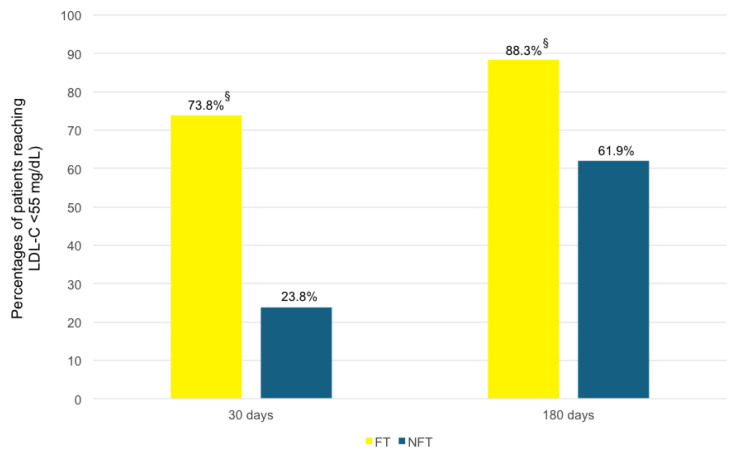
Percentages of patients that reached the recommended LDL-C target (<55 mg/dL) in the “PCSK9i NFT” and “PCSK9i FT” groups at 30 days and 180 days. The percentage of patients in the “PCSK9i FT” group that reached the recommended LDL-C target (<55 mg/dL) at 30 days was 73.8% versus 23.8% in the “PCSK9i NFT” group, whereas the percentage of patients in the “PCSK9i FT” group that reached the recommended LDL-C target (<55 mg/dL) at 180 days was 88.3% versus 61.9% in the “PCSK9i NFT” group. FT, fast track; LDL-C, low-density lipoprotein cholesterol; NFT, non-fast track. ^§^ *p* < 0.001 versus the “PCSK9i NFT” group.

The percentages of patients in the “PCSK9i FT” group who reached the recommended LDL-C target (<55 mg/dL) at 30 days and 180 days were 73.8% and 88.3%, respectively, versus percentages of 23.8% and 61.9% in the control group (*p*-value < 0.001; Figure 4).

During hospitalization, one death was detected in the control group. During the follow-up period, one death, two myocardial infarctions, and one stent thrombosis were detected in the control group, whereas one myocardial infarction and two ischemia-driven revascularizations were detected in the “PCSK9i fast track” group. No adverse reactions due to early PCSK9i administration (i.e., injection-site reaction or pain, fatigue, headache, influenza, and illness) were detected. All MACEs, adverse reactions, and events of therapy discontinuation occurring during hospitalization and the follow-up period are summarized in Table 3.

## 4. Discussion

The primary aim of the “PCSK9i fast track” approach is to achieve, as early as possible, the therapeutic target of LDL-C (<55 mg/dL) in the highest percentage of patients. In fact, the strong association between high LDL-C levels and the risk of atherosclerotic cardiovascular disease progression is well established, as is the link between LDL-C reduction and decreased risk of MACEs [16].

To our knowledge, this retrospective single-center study is the first to compare the efficacy of the “PCSK9i fast track” strategy versus the “stepwise” guideline-driven LLT (that includes the addition of PCSK9i during the follow-up, according to the 2019 ESC guidelines), in terms of lowering LDL-C and non-HDL-C, in a “real-world” population of ACS patients. Indeed, previous randomized clinical trials had compared the early administration of PCSK9is with a placebo alone in addition to high-dosage statin and ezetimibe therapy in terms of lipid-lowering power and stabilization of soft atherosclerotic plaques [9,10,11,12,13].

Our study also demonstrates the feasibility of the “PCSK9i fast track” strategy in a “real-world” setting. A previous study investigated the change in PCSK9i prescription trends since the publication of the 2019 ESC guidelines. The authors chose to extend the indication for the PCSK9i prescription to LLT-naïve patients with LDL-C > 140 mg/dL, as authorized by the AIFA in 2022. The results showed a significant increase in the percentage of subjects receiving triple LLT (statin + ezetimibe + PCSK9i) during the observation period, from 3% at the study’s start to 27% at its end, with very high compliance with this treatment strategy [17]. Nevertheless, the PCSK9i was not truly administered according to a “fast track” strategy in that study because therapy began at discharge from ACS hospitalization, whereas in our study the PCSK9i was administered within 48 h of ACS diagnosis. The early administration of the first dose was achievable due to our knowledge of the LDL-C baseline value within 24 h of admission, which allowed us to promptly request the PCSK9i from our hospital’s pharmacy and initiate therapy within 48 h of admission.

We found that the “PCSK9i fast track” approach is associated with significantly lower values of TC, TG, LDL-C, and non-HDL-C and higher HDL-C values at 30 and 180 days of follow-up compared with the “stepwise” guideline-driven LLT (Figure 2 A and B). At 30 and at 180 days, the difference in the mean percentage change of LDL-C from baseline to follow-up was statistically significant between the “PCSK9i FT” and “PCSK9i NFT” groups (Figure 3). The recommended LDL-C target (<55 mg/dL) at 30 and 180 days was achieved by 73.8% and 88.3% of patients in the “PCSK9i FT” group versus 23.8% and 61.9% in the control group, respectively (Figure 4). Our results are consistent with previous clinical studies.

In the EVO-PACS study, the administration of evolocumab, within 72 h in non-ST-elevation myocardial infarction (NSTEMI) patients and within 24 h in ST-segment elevation myocardial infarction (STEMI) patients, significantly reduced LDL-C compared with atorvastatin 40 mg therapy [12]. The mean percentage change from baseline at 8 weeks of follow-up was −40.7% (95% confidence interval: −45.2 to −36.2; *p*-value < 0.001). LDL-C levels < 55 mg/dL were achieved at week 8 by 91.3% of patients in the evolocumab group versus 37.6% in the placebo group. In the HUYGENS study, considering a longer follow-up period (50 weeks), the initiation of evolocumab within a maximum of 7 days after hospital admission in NSTEMI patients reduced LDL-C by 80% compared with 39% in controls on maximally tolerated statins alone (*p*-value between groups < 0.001) [13]. A significantly greater proportion of patients treated with evolocumab achieved an on-treatment LDL-C < 55 mg/dL (86.4% vs. 20.0%, *p*-value < 0.001). In the EPIC-STEMI study, the first injection of PCSK9i was given before the primary PCI regardless of baseline LDL-C levels [10]. Even in this case, LDL-C decreased more significantly in the PCSK9i group (alirocumab) compared with the sham-control (72.9% vs. 48.1%), with a mean between-group difference of −22.3% (*p*-value < 0.001), at 8 weeks of follow-up. A higher percentage of patients achieved an LDL-C value < 55 mg/dL in the alirocumab group compared with the placebo group (92.1% vs. 56.7%, *p*-value < 0.001). In all three studies, non-HDL-C levels were significantly lower in the PCSK9i group than in the placebo group at hospital discharge and at 30 days. Importantly, none of these trials included the “stepwise” addition of a PCSK9i in the control group.

Although the “PCSK9i fast track” strategy resulted in a significantly higher lipid-lowering effect compared with the “stepwise” guideline-driven LLT, we did not find statistically relevant differences in outcomes in the two investigated groups. Consistently, in a recent randomized clinical trial of similar size comparing three months of LLT with the PCSK9i followed by conventional LLT and twelve months of conventional LLT alone, no significant differences were detected in the primary endpoint, including the composite of all-cause death, myocardial infarction, stroke, unstable angina, and ischemia-driven revascularization [18]. The small size of our study and the short duration of the follow-up period (180 days) could explain this result. In fact, a more recent metanalysis encompassing 2896 ACS patients demonstrated the efficacy of the “PCSK9i fast track” strategy in reducing the incidence of MACEs compared with statin monotherapy when a large population and a long follow-up period were considered [19].

In the future, it is conceivable that the “PCSK9i fast track” strategy may be shown to play a pivotal role beyond lowering LDL-C in ACS patients. Indeed, the “PCSK9i fast track” strategy has been demonstrated to significantly stabilize non-culprit atherosclerotic coronary plaques by increasing fibrous cap thickness [11,13]. The concomitant decrease in atheroma volume and plaque lipid content and the increase in fibrous cap thickness led to a significant reduction in MACEs in a sub-analysis of the PACMAN-AMI study [20]. Furthermore, PCSK9is have been shown to decrease the inflammatory state of the myocardium [21], to have an inhibitory effect on platelet aggregation inside the coronary arteries [22], and to improve the survival of cardiac myocytes against reperfusion injury [23]. More recently, in patients with STEMI, a significant increase in PCSK9 was observed from 24 to 48 h after PCI. Interestingly, PCSK9 after 48 h was significantly associated with intramyocardial hemorrhage, microvascular obstruction, and infarct size as well as worse subsequent clinical outcomes [24]. All these findings allow us to speculate that PCSK9 may represent a potentially valuable biomarker for the risk stratification of patients with STEMI and that the “PCSK9i fast track” strategy may be considered a tool, independently of starting LDL-C values, to fight myocardial ischemia–reperfusion injury [25]. The data from ongoing large randomized trials evaluating the effects of the “PCSK9i fast track” strategy on MACE reduction, myocardial salvage, and left ventricular remodeling incidence by magnetic resonance imaging could further support this hypothesis [26,27].

Lastly, our data demonstrate that the “fast track” use of both alirocumab and evolocumab has a favorable safety profile and is well tolerated compared with the “stepwise” guideline-driven LLT. These results are in line with the current literature. The percentage of patients who experienced adverse events, serious adverse events, and adverse events leading to study drug discontinuation was similar between groups in the EVOPACS study [12]. Consistently, in the EPIC-STEMI trial, there were no reported local injection site reactions, allergic reactions, or intracranial hemorrhages [10].

## 5. Limitations

There are some limitations of our study that deserve consideration. First, as mentioned in the Discussion, the study size was modest, and the study duration was short. Moreover, our study is based on a retrospective analysis of an ACS population treated in our Cardiology Division during two different periods. Second, the “PCSK9i FT” and “PCSK9i NFT” groups differed significantly in the prevalence of familiarity for CAD and arterial hypertension; furthermore, they significantly differed in age, TC, and LDL-C. This lack in homogeneity may be explained by the trend to more aggressively treat younger patients with a higher number of CV risk factors and elevated lipid values. Third, although there was a clear reduction in LDL-C and non-HDL-C levels in the “PCSK9i FT” population, our trial was not designed to evaluate clinical outcomes. Indeed, a key limitation of our study is the low number of MACE events, which prevents a more in-depth analysis to assess potential confounders such as age and hypertension. Future studies with larger cohorts and a higher number of events are needed to better evaluate if these factors potentially influence the interpretation of our findings. Fourth, although PCSK9is rapidly reduce LDL-C levels (within days) [28], lipid levels were first measured 30 days after the first administration of the study drug; thus, we could not capture earlier effects of both alirocumab and evolocumab in this study setting. Fifth, we did not measure apolipoprotein B (apoB) and lipoprotein(a) values in our population but, more generally, non-LDL-C, because these parameters were not measured in most of the enrolled population during their in-hospital stay. Both apoB and lipoprotein(a) have an important prognostic role in the ACS population. In fact, it has been previously demonstrated that MACEs increased across baseline apoB strata [29]. Alirocumab has been shown to reduce MACEs across all strata of baseline apoB, with larger absolute reductions in patients with higher baseline levels. Lower apoB values were associated with a decreased risk of MACEs, even after accounting for the achieved LDL-C or non–HDL-C, indicating that apoB provides incremental information. In particular, the achievement of apoB levels as low as ≤35 mg/dL has been demonstrated to reduce the lipoprotein-attributable residual risk after ACS [30]. Similarly, lipoprotein(a) is a risk factor for CV events and modifies the benefit of PCSK9is. Indeed, in patients with recent ACS and LDL-C near 70 mg/dL on optimized statin therapy, PCSK9 inhibition provides an incremental clinical benefit only when the lipoprotein(a) concentration is at least mildly elevated (>13.7 mg/dL) [31].

## 6. Conclusions

The use of the “PCSK9i fast track” strategy in patients admitted to our hospital with ACS was associated with significantly lower TC, LDL-C, TG, and non-HDL-C values compared with the “stepwise” guideline-driven LLT at 30 and 180 days of follow-up. At 30 and 180 days of follow-up, the difference in the mean percentage change of LDL-C from baseline to follow-up was significantly higher in the “PCSK9i FT” group compared with the “PCSK9i NFT” group. The percentages of patients in the “PCSK9i FT” group who reached the recommended LDL-C target (<55 mg/dL) were preminent compared with the “PCSK9i NFT” group, both at 30 and 180 days of follow-up. The “PCSK9i fast-track” strategy was not associated with new adverse effects. There were no statistically relevant differences in the incidence of MACEs between the “PCSK9i FT” and “PCSK9i NFT” groups.

Our results further support the efficacy, feasibility, and safety of using the “PCSK9i fast track” strategy during hospitalization for ACS. This pharmacological strategy may account for the more rapid achievement of stabilization of non-culprit atherosclerotic coronary plaques and allow us to exploit the pleiotropic effects of PCSK9is in terms of anti-inflammatory properties, the inhibition of platelet aggregation, and the improvement of cardiac myocyte survival.

## Figures and Tables

**Table 1:** Statistical analysis for categorical variables.

Variable	PCSK9i FT	PCSK9i NFT	*p*-Value
Sex, n (%)			0.25
Male	47 (73.4)	50 (82.0)	
Female	17 (26.6)	11 (18.0)	
Familiarity, n (%)			0.02
no	35 (54.7)	46 (75.4)	
yes	29 (45.3)	15 (24.6)	
Diabetes mellitus, n (%)			0.21
no	54 (84.4)	46 (75.4)	
yes	10 (15.6)	15 (24.6)	
Smoker, n (%)			0.82
no	19 (29.7)	20 (32.8)	
current	43 (67.2)	40 (65.6)	
former	2 (3.1)	1 (1.6)	
Arterial Hypertension, n (%)			0.02
no	11 (17.2)	22 (36.1)	
yes	53 (82.8)	39 (63.9)	
Diagnosis, n (%)			0.02
Unstable angina	4 (6.2)	0 (0.0)	
NSTEMI	7 (10.9)	1 (1.7)	
STEMI	53 (82.8)	57 (98.3)	
LLT naive, n (%)			0.23
yes	10 (15.3)	16 (25.4)	
no	55 (84.6)	47 (74.6)	
CASS score, n (%)			0.86
0	1 (1.6)	1 (1.8)	
1	23 (35.9)	18 (31.6)	
2	20 (31.2)	22 (38.6)	
3	20 (31.2)	16 (28.1)	
Proximal segment, n (%)			0.47
no	25 (39.1)	19 (32.8)	
yes	39 (60.9)	39 (67.2)	
OCT, n (%)			0.3
no	59 (92.2)	56 (96.6)	
yes	5 (7.8)	2 (3.4)	
IVUS, n (%)			0.2
no	55 (85.9)	54 (93.1)	
yes	9 (14.1)	4 (6.9)	

In this table are reported the characteristics of the two study groups as categorical variables. Categorical variables are expressed as a percentage and were compared using the Chi-Square test or Fisher’s exact test. A *p*-value of <0.05 was considered significant. Bonferroni’s correction was used for multiple hypothesis correction if necessary. CASS, Coronary Artery Surgery Study; IVUS, Intra-Vascular Ultra-Sound; LLT, lipid-lowering therapy; NSTEMI, non-ST-elevation myocardial infarction; OCT, Optical Coherence Tomography; STEMI, ST-segment elevation myocardial infarction.

**Table 2:** Statistical analysis for continuous variables.

Variable	PCSK9i FT			PCSK9i NFT			
	N	Mean	SD	N	Mean	SD	*p*-Value
BMI	64	28.028	4.589	58	28.444	4.642	0.560
Age	64	57.094	10.033	61	62.180	11.275	0.008
Stent total lenght	64	70.375	41.177	57	68.579	40.008	0.780
Stent maximum diameter	64	3.523	0.654	58	3.759	0.647	0.032
LVEF at admission	63	43.714	7.408	60	43.250	6.964	0.860
LVEF at discharge	63	47.429	7.411	60	46.017	9.438	0.550
Glucose blood level at admission	63	119.429	67.956	60	132.333	69.353	0.220
Glucose blood level at discharge	63	92.889	21.350	60	105.983	50.613	0.400
TC day 0	64	200.969	42.636	62	192.806	47.382	0.370
LDL-C day 0	64	128.444	40.112	62	121.526	43.525	0.310
TG day 0	64	133.719	56.501	62	135.113	62.916	0.950
HDL day 0	64	45.781	11.182	62	44.258	10.504	0.410
Non-HDL-C day 0	64	155.188	44.272	62	148.548	46.656	0.530
TC day 30	53	98.208	27.862	57	156.246	39.723	
LDL-C day 30	60	41.563	27.573	62	85.590	35.896	<0.001
TG day 30	53	90.679	39.675	57	117.404	57.631	0.003
HDL day 30	53	42.830	10.493	57	45.544	10.712	0.140
Non-HDL-C day 30	55	53.364	28.731	57	111.491	38.068	<0.001
TC day 180	51	92.588	23.417	53	122.226	36.227	<0.001
LDL-C day 180	59	29.617	21.026	61	59.049	32.405	<0.001
TG day 180	47	89.319	33.620	51	110.882	47.908	0.001
HDL day 180	49	48.653	11.300	53	43.453	11.150	0.019
Non-HDL-C day 180	52	44.962	22.913	54	77.315	35.193	<0.001

In this table are reported the characteristics of the two study groups as continuous variables. Continuous variables are expressed as the mean and standard deviation (SD). The data distribution was tested for normality through the Shapiro–Wilk test. An unpaired Student’s *t*-test or Wilcoxon rank-sum test, as required, was used for comparison between the two groups. BMI, body mass index; HDL-C, high-density lipoprotein cholesterol; LDL-C, low-density lipoprotein cholesterol; LVEF, left ventricular ejection fraction; non-HDL-C, non-high-density lipoprotein cholesterol; TC, total cholesterol; TG, triglycerides.

**Table 3:** MACEs, adverse reactions, and events of therapy discontinuation occurring during hospitalization and the follow-up period.

	MACEs, Adverse Reactions, and Discontinuation of Therapy	PCSK9i FT	PCSK9i NFT
In-hospital	Death	0	1
	Myocardial infarction	0	0
	Ischemia-driven revascularization	0	0
	Stent thrombosis	0	0
	Adverse reactions (injection-site reaction or pain, fatigue, headache, influenza, and illness)	0	0
	Discontinuation of therapy	0	0
During follow-up	Death	0	1
	Myocardial infarction	1	2
	Ischemia-driven revascularization	1	0
	Stent thrombosis	0	1
	Adverse reactions (injection-site reaction or pain, fatigue, headache, influenza, and illness)	0	0
	Discontinuation of therapy	0	0

In this table are reported the crude values regarding MACEs, adverse reactions, and events of therapy discontinuation occurring during hospitalization and the follow-up period in the two study groups. MACEs, major adverse cardiovascular events.

## Data Availability

The data are available from the corresponding author on reasonable request.

## References

[1] van der Bijl P., Abou R., Goedemans L., Gersh B.J., Holmes D.R., Marsan N.A., Delgado V., Bax J.J. (2020). Left Ventricular Post-Infarct Remodeling: Implications for Systolic Function Improvement and Outcomes in the Modern Era. JACC Heart Fail..

[2] Sabatine M.S., De Ferrari G.M., Giugliano R.P., Huber K., Lewis B.S., Ferreira J., Kuder J.F., Murphy S.A., Wiviott S.D., Kurtz C.E. (2018). Clinical Benefit of Evolocumab by Severity and Extent of Coronary Artery Disease: Analysis From FOURIER. Circulation.

[3] Schwartz G.G., Steg P.G., Szarek M., Bhatt D.L. (2018). Alirocumab and Cardiovascular Outcomes after Acute Coronary Syndrome. N. Engl. J. Med..

[4] Laufs U., Catapano A.L., De Caterina R., Schiele F., Sionis A., Zaman A., Jukema J.W. (2023). The effect of the 2019 ESC/EAS dyslipidaemia guidelines on low-density lipoprotein cholesterol goal achievement in patients with acute coronary syndromes: The ACS EuroPath IV project. Vasc. Pharmacol..

[5] Ferlini M., Munafò A., Varbella F., Delnevo F., Solli M., Trabattoni D., Piccaluga E., Cardile A., Canova P., Rossini R. (2024). Achievement of target LDL-cholesterol level in patients with acute coronary syndrome undergoing percutaneous coronary intervention: The JET-LDL registry. Int. J. Cardiol..

[6] Krychtiuk K.A., Ahrens I., Drexel H., Halvorsen S., Hassager C., Huber K. (2022). Acute LDL-C reduction post ACS: Strike early and strike strong: From evidence to clinical practice. A clinical consensus statement of the Association for Acute CardioVascular Care (ACVC), in collaboration with the European Association of Preventive Cardiology (EAPC) and the European Society of Cardiology Working Group on Cardiovascular Pharmacotherapy. Eur. Heart J. Acute Cardiovasc. Care.

[7] Fogacci F., Giovannini M., Grandi E., Imbalzano E. (2022). Management of High-Risk Hypercholesterolemic Patients and PCSK9 Inhibitors Reimbursement Policies: Data from a Cohort of Italian Hypercholesterolemic Outpatients. J. Clin. Med..

[8] Byrne R.A., Rossello X., Coughlan J.J., Barbato E., Berry C., Chieffo A., Claeys M.J., Dan G.A., Dweck M.R., Galbraith M. (2023). 2023 ESC Guidelines for the management of acute coronary syndromes. Eur. Heart J..

[9] Leucker T.M., Blaha M.J., Jones S.R., Vavuranakis M.A., Williams M.S., Lai H., Schindler T.H., Latina J., Schulman S.P., Gerstenblith G. (2020). Effect of Evolocumab on Atherogenic Lipoproteins During the Peri- and Early Postinfarction Period: A Placebo-Controlled, Randomized Trial. Circulation.

[10] Mehta S.R., Pare G., Lonn E.M., Jolly S.S., Natarajan M.K., Pinilla-Echeverri N., Schwalm J.D., Sheth T.N., Sibbald M., Tsang M. (2022). Effects of routine early treatment with PCSK9 inhibitors in patients undergoing primary percutaneous coronary intervention for ST-segment elevation myocardial infarction: A randomised, double-blind, sham-controlled trial. EuroIntervention.

[11] Räber L., Ueki Y., Otsuka T., Losdat S., Häner J.D., Lonborg J., Fahrni G., Iglesias J.F., van Geuns R.J., Ondracek A.S. (2022). Effect of Alirocumab Added to High-Intensity Statin Therapy on Coronary Atherosclerosis in Patients With Acute Myocardial Infarction: The PACMAN-AMI Randomized Clinical Trial. JAMA.

[12] Koskinas K.C., Windecker S., Pedrazzini G., Mueller C., Cook S., Matter C.M., Muller O., Häner J., Gencer B., Crljenica C. (2019). Evolocumab for Early Reduction of LDL Cholesterol Levels in Patients with Acute Coronary Syndromes (EVOPACS). J. Am. Coll. Cardiol..

[13] Nicholls S.J., Kataoka Y., Nissen S.E., Prati F., Windecker S., Puri R., Hucko T., Aradi D., Herrman J.R., Hermanides R.S. (2022). Effect of Evolocumab on Coronary Plaque Phenotype and Burden in Statin-Treated Patients Following Myocardial Infarction. JACC Cardiovasc. Imaging.

[14] Mach F., Baigent C., Catapano A.L., Koskinas K.C., Casula M., Badimon L., Chapman M.J., De Backer G.G., Delgado V., Ference B.A. (2020). 2019 ESC/EAS Guidelines for the management of dyslipidaemias: Lipid modification to reduce cardiovascular risk. Eur. Heart J..

[15] Serruys P.W., Onuma Y., Garg S., Sarno G., van den Brand M., Kappetein A.P., Van Dyck N., Mack M., Holmes D., Feldman T. (2009). Assessment of the SYNTAX score in the Syntax study. EuroIntervention.

[16] Ference B.A., Ginsberg H.N., Graham I., Ray K.K., Packard C.J., Bruckert E., Hegele R.A., Krauss R.M., Raal F.J., Schunkert H. (2017). Low-density lipoproteins cause atherosclerotic cardiovascular disease. 1. Evidence from genetic, epidemiologic, and clinical studies. A consensus statement from the European Atherosclerosis Society Consensus Panel. Eur. Heart J..

[17] Muccioli S., Giglio C., Annibali G., Cerutti E., Civera S., Casati R., Delnevo F., De Rosa C., Bongioanni S., Colopi M. (2022). The importance of intensive lipid-lowering therapy after acute coronary syndrome: Changing the paradigm to improve the achievement of targets. Eur. Heart J..

[18] Yamashita S., Sakamoto A., Shoji S. (2023). Feasibility of Short-Term Aggressive Lipid-Lowering Therapy with the PCSK9 Antibody in Acute Coronary Syndrome. J. Cardiovasc. Dev. Dis..

[19] Deng Y., Ma Y., Zhang Y., Gao J., Sun X., He S., Zhu L., Zhang J. (2024). The impact of early in-hospital use of PCSK9 inhibitors on cardiovascular outcomes in acute coronary syndrome patients: A systematic review and meta-analysis. Int. J. Cardiol..

[20] Biccirè F.G., Häner J., Losdat S., Ueki Y., Shibutani H., Otsuka T., Kakizaki R., Hofbauer T.M., van Geuns R.J., Stortecky S. (2023). Concomitant Coronary Atheroma Regression and Stabilization in Response to Lipid-Lowering Therapy. J. Am. Coll. Cardiol..

[21] Wu C., Lin D., Ji J., Jiang Y., Jiang F., Wang Y. (2023). PCSK9 Inhibition Regulates Infarction-Induced Cardiac Myofibroblast Transdifferentiation via Notch1 Signaling. Cell Biochem. Biophys..

[22] Franchi F., Ortega-Paz L., Rollini F., Been L., Rivas A., Maaliki N., Zhou X., Pineda A.M., Suryadevara S., Soffer D. (2023). Impact of evolocumab on the pharmacodynamic profiles of clopidogrel in patients with atherosclerotic cardiovascular disease: A randomised, double-blind, placebo-controlled study. EuroIntervention.

[23] Xu Q., Zhao Y.M., He N.Q., Gao R., Xu W.X., Zhuo X.J., Ren Z., Wu C.Y., Liu L.S. (2023). PCSK9: A emerging participant in heart failure. Biomed. Pharmacother.=Biomed. Pharmacother..

[24] Tiller C., Holzknecht M. (2024). Association of Circulating PCSK9 with Ischemia-Reperfusion Injury in Acute ST-Elevation Myocardial Infarction. Circ. Cardiovasc. Imaging.

[25] Benedetti A. (2024). Is PCSK9 the Key Player in the Ischemia-Reperfusion Match?. Circ. Cardiovasc. Imaging.

[26] Gao J., Liu J.Y., Lu P.J., Xiao J.Y., Gao M.D., Li C.P., Cui Z., Liu Y. (2021). Effects of Evolocumab Added to Moderate-Intensity Statin Therapy in Chinese Patients With Acute Coronary Syndrome: The EMSIACS Trial Study Protocol. Front. Physiol..

[27] Xia J., Wang X., Zhou J., Wang D., Pang Y., Xu X., Sang Z., Zhang Y., Zhang J., Wu S. (2022). Impact of early PCSK9 inhibitor treatment on heart after percutaneous coronary intervention in patients with STEMI: Design and rationale of the PERFECT II trial. Front. Cardiovasc. Med..

[28] Dias C.S., Shaywitz A.J., Wasserman S.M., Smith B.P., Gao B., Stolman D.S., Crispino C.P., Smirnakis K.V., Emery M.G., Colbert A. (2012). Effects of AMG 145 on low-density lipoprotein cholesterol levels: Results from 2 randomized, double-blind, placebo-controlled, ascending-dose phase 1 studies in healthy volunteers and hypercholesterolemic subjects on statins. J. Am. Coll. Cardiol..

[29] Johannesen C.D.L., Mortensen M.B., Langsted A., Nordestgaard B.G. (2021). Apolipoprotein B and Non-HDL Cholesterol Better Reflect Residual Risk Than LDL Cholesterol in Statin-Treated Patients. J. Am. Coll. Cardiol..

[30] Hagström E., Steg P.G., Szarek M., Bhatt D.L., Bittner V.A., Danchin N., Diaz R., Goodman S.G., Harrington R.A., Jukema J.W. (2022). Apolipoprotein B, Residual Cardiovascular Risk After Acute Coronary Syndrome, and Effects of Alirocumab. Circulation.

[31] Schwartz G.G., Szarek M., Bittner V.A., Diaz R., Goodman S.G., Jukema J.W., Landmesser U., López-Jaramillo P., Manvelian G., Pordy R. (2021). Lipoprotein(a) and Benefit of PCSK9 Inhibition in Patients With Nominally Controlled LDL Cholesterol. J. Am. Coll. Cardiol..

